# Comparison of oocyte count, fertilization, and pregnancy rates in adenomyosis patients undergoing In Vitro Fertilization with short and long protocol controlled ovarian stimulation – Restospective study

**DOI:** 10.1016/j.amsu.2022.104620

**Published:** 2022-09-16

**Authors:** Tjahyadi Dian, Pribadi Adhi, Rizqi Subhan Darojat Ar, Nisa Aisyah Shofiatun, Djuwantono Tono

**Affiliations:** a Department of Obstetrics and Gynecology, Faculty of Medicine, Universitas Padjadjaran - Dr. Hasan Sadikin Hospital, Bandung, Indonesia; b Bandung Fertility Center, Limijati Women and Children Hospital, Bandung, Indonesia

**Keywords:** Adenomyosis, Fertility, IVF Protocol

## Abstract

**Introduction:**

Adenomyosis (benign gynecological disease) is an endometrial stromal tissue condition that invades the myometrium of the uterus. The administration of a gonadotropin-releasing hormone (GnRH) analog before the IVF (In Vitro Fertilization) cycle significantly increases the chances of getting pregnant in adenomyosis patient with infertile condition. There is no best protocol consensus for adenomyosis for now. This study plans to compare the outcomes of long-protocol and short-protocol of IVF in adenomyosis patients who have undergone surgery and treatment with GnRH analogs.

**Method:**

This study is a retrospective study with a comparative method. The study was conducted at the IVF Aster Clinic and the IVF Bandung Fertility Center by reviewing retrospective data from 2015 to 2021. Patients who have been diagnosed with adenomyosis will undergo IVF procedure with a long-protocol and short-protocol pretreatment. Parameters observed were oocyte count, fertilization, and pregnancy rate.

**Results:**

Fifty-eight patients were included. There was a significant difference in the oocytes count and the pregnancy rate in short and long groups with p value of less than 0.05, while there was no significant difference in fertilization rate with p value of greater than 0.05.

**Conclusion:**

There were differences in the oocytes count and pregnancy rates in the short and long protocol groups in adenomyosis patients. There was no difference in fertilization rate in the short and long protocol groups in adenomyosis patients.

## Highlights

• The number of oocytes and pregnancy rates were significantly difference in the short and long protocols.
• The fertilization rates was not significantly difference in the short and long protocols.
• Long protocols of GnRH agonists produced better effects than short protocols of GnRH antagonists, as indicated by clinical pregnancy rates (higher β-hCG levels).

## 1 Introduction

Adenomyosis (benign gynecological disease) is an endometrial stromal tissue condition that invades the myometrium of the uterus. The triad symptoms of adenomyosis are abnormal uterine bleeding, enlarged-tender uterus, and dysmenorrhea. Recently, cases of adenomyosis have been increasingly found in infertile patients which undergo in vitro fertilization (IVF) proccess [1]. The incidence of adenomyosis globally is 1.03% or 28.9 per 10,000 women/year, while in Indonesia is still not well measured, but the incidence is 2.39%–11.7% from several different literatures [2,3].

There is a relationship between adenomyosis and disruption of the natural process of conception [4,5]. The rate of early miscarriage is two times higher in adenomyosis patients. Adenomyosis patients who undergo IVF results in a low live birth rate. It is suspected that there is a disturbance in the embryo invasion and the placenta formation [6].

Adenomyomectomy is the procedures that can be performed in cases of adenomyosis. However, fewer pregnancy rates were found in patients undergoing adenomyomectomy (18.2%) than in IVF (In Vitro Fertilization) procedures (38.8%) [7]. Several study showed that the administration of a gonadotropin-releasing hormone (GnRH) analog before the IVF cycle significantly increases the chances of getting pregnant in adenomyosis patient with infertile condition. Various ways to increase the chances of pregnancy in adenomyosis cases undergoing IVF are combining GnRH analogs with frozen-thawed embryo transfer (FET) [8].

Long-protocol IVF with a GnRH analog is a popular regimen in IVF for adenomyosis patients. The process of apoptosis in women with adenomyosis was significantly induced by administration of GnRH agonists in reducing of angiogenesis and inflammatory reactions. The longer protocol is more widely used to increase pregnancy rates in women with adenomyosis [6]. Short protocol IVF with GnRH antagonists was suggested as the protocol alternative. Because a long protocol of GnRH agonists can induce an initial flare-up effect. The short protocol of GnRH antagonists was given to avoid excessive pituitary suppression in the event of poor ovarian response to the long protocol. There is no consensus on the best protocol for adenomyosis [8]. This study plans to compare the outcomes of long-protocol IVF and short-protocol IVF in adenomyosis patients who have surgery and treatment with GnRH analogs for 3 months. Parameters observed were oocyte count, fertilization rate, and pregnancy rate.

## 2 Material and methods

The study was approved by the Research Ethics Committee of Dr. Hasan Sadikin Hospital, Bandung, Indonesia, Nomor: LB.02.01/X.6.5/159/2022. All patients in this study were examined and adhered to the ethical standards set out in the 1964 Declaration of Helsinki. This study had been also registry in www.researchregistry.com with research registry unique identifying number researchregistry8088. This paper had been written used STROCSS 2021 guideline [9].

This study aimed to determine differences in oocyte count, fertilization rate, and pregnancy rate of IVF patients in short and long protocol. This study was a retrospective study with a comparative method. The comparative method is research that compares the variables related to each other by stating the differences or similarities that occur. The comparative research method is ex post facto.

The subjects of this study were all patients with adenomyosis who underwent IVF procedures at the Aster and Bandung Fertility Clinics during 2015–2021. Secondary data will be used in this study. The size of the sample was determined based on the formula for unpaired numerical categorical analysis research. Based on these calculations, the minimum number of samples for each group is 26 sample, then 10% is added (in case of lost follow-up patients), so that the total number of samples needed is 29 people for each group.

The inclusion criteria were patients with adenomyosis, who had undergone surgery for adenomyosis and received GnRH agonist therapy for 3 months, underwent IVF with a short protocol and a long protocol, aged 20–40 years, level AMH (Anti‐Müllerian hormone) was 2.2–4, and AFC (antral follicle count) of 5–15. The ultrasound criteria used to confirm the diagnosis of adenomyosis are the Morphological Uterus Sonographic Assessment (MUSA) criteria conducted by the fertility and endocrine consultant [10].

The type of surgery performed on focal type adenomyosis patients was resection surgery while the diffuse type adenomyosis was osada surgery. The most frequent complication of surgery is bleeding, all patients have received blood transfusions so that they return home in good condition. All patients in this study were patients with primary infertility. Several other infertility factors that assessed were male factors, but we did not include it in the study because during ICSI we selected the best sperm quality.

While the exclusion criteria were patients with ovulation disorders, adenomyosis patients who had not undergone surgery and GnRH agonist therapy, and patients who did not completely follow the IVF process. The matching procedure was carried out in selecting groups on the independent variables based on age, level of AMH, and count of AFC patients in each short and long protocol group.

In long protocols, the patients were administered GnRH agonists in the luteal phase of the previous cycle and continued until hCG administration. This protocol will result in the intrinsic activity of agonist compounds causing pituitary down-regulation, which is preceded by an early stimulation phase known as the flare effect. GnRH antagonists can be given any time during to prevent premature LH surge [10]. Injection of a GnRH agonist (Buserelin 0.5 mg) is started 10–14 days before gonadotropin administration. The administration of GnRH agonists is usually initiated in the middle luteal phase. Gonadotropin injection was started after down-regulation was achieved, and the dose of the GnRH agonist was reduced to 0.2 mg. Stimulation with gonadotropins was continued until the follicle diameter reached 17–18 mm in at least three follicles [10].

In the short protocol, injections of GnRH antagonists were administered regularly (0.25 mg per day starting on day 6 or 7 stimulation) or flexible (0.25 mg per day if the largest follicle was 14–15 mm). A single dose of 3 mg GnRH antagonist can be injected on the seventh and eighth days of stimulation with or without the addition of an oral contraceptive pill [10].

In each group, the oocyte count, fertilization rate, and pregnancy rate will be calculated. The oocytes count was evaluated at the time of oocyte collection from the ovary by the fertility and endocrine consultant. The fertilization rate is the percentage of embryos obtained after ICSI (intra-cytoplasmic sperm injection). Meanwhile, the pregnancy rate was measured from β-hCG levels on day 16 after ovum pick up used fresh embryo.

The normality test used was Kolmogorov Smirnov. The significance test in comparing the characteristics of the two groups used the unpaired *t*-test (normally distributed) and the Mann Whitney (not normally distributed). While the significance test on categorical data used Chi-square with alternative Exact Fisher's and Kolmogorov Smirnov test. The statistical analyses were performed by SPSS™ (24.0.0).

## 3 Results

There were 58 patients in this study, the average age of the patients was 34.14 ± 4.24 years old, with the most recent education being strata-1 as much as 30 (51.7%). A total of 22 people (30.1%) work as housewives, while others work as employees, entrepreneurs, and civil servants. The average body mass index of patients was 23.60 ± 2.71, and the level of AMH patients had an average of 2.67 ± 2.46 (Table 1). Most of them were of focal type adenomyosis (93.1%). Based on Table 1, there were no differences in characteristics between groups of patients who underwent short or long protocols (p value > 0.05).

**Table 1:** Background characteristics of the study population.

Variable	N = 58	Group		P Value
		Short Protocol	Long Protocol	
		N = 29	N = 29	
**Age (years)**				0.903
Mean ± Std	34.14 ± 4.24	34.21 ± 4.48	34.07 ± 4.07	
Median	34.00	34.00	35.00	
Range (min-max)	19.00 (24.00–44.00)	19.00 (25.00–44.00)	14.00 (27.00–41.00)	
**Study**				0.568
High School	5 (8.6%)	2 (6.9%)	3 (10.3%)	
Diploma	16 (27.6%)	8 (27.6%)	8 (27.6%)	
Bachelor	30 (51.7%)	17 (58.6%)	13 (44.8%)	
Post Graduate	7 (12.1%)	2 (6.9%)	5 (10.3%)	
**Work**				
Housewifes	17 (29.3%)	9 (31.0%)	8 (27.6%)	0.182
Employee	20 (34.5%)	6 (20.7%)	14 (48.3%)	
Entrepreneur	3 (5.2%)	2 (6.9%)	1 (3.4%)	
Civil Servant	15 (25.9%)	11 (37.9%)	4 (13.8%)	
others	3 (5.2%)	1 (3.4%)	2 (6.8%)	
**BMI**				0.157
Mean ± Std	23.60 ± 2.71	23.10 ± 2.89	24.11 ± 2.46	
Median	23.40	23.10	24.10	
Range (min-max)	13.20 (18.30–31.50)	13.20 (18.30–31.50)	8.90 (19.60–28.50)	
**AMH**				0.756
Mean ± Std	2.67 ± 2.46	2.95 ± 3.06	2.40 ± 1.67	
Median	1.92	1.80	1.97	
Range (min-max)	11.94 (0.16–12.10)	11.94 (0.16–12.10)	7.70 (0.72–8.42)	
**Adenomyosis type**				1.000
Focal	54 (93.1%)	27 (93.1%)	27 (93.1%)	
Diffuse	4 (0.9%)	2 (6.9%)	2 (6.9%)	

Table 2 describes the differences in oocytes, fertilization, and pregnancy rate in the short and long groups. In the short group, the average number of oocytes in the short group was 5.60 ± 3.5, while in the long group, the average number of oocytes was 10.48 ± 6.66. Based on the results of both statistical tests, it was found that there was a statistically significant difference between the variable number of oocytes in the short and long groups. Meanwhile, at the fertilization rate, there was no significant difference between the short and long groups (p-value > 0.05).

**Table 2:** Comparison mean, median in between oocyte count, fertility rate, and pregnancy rate in the short and long protocol.

Variable	Group		P Value
	Short Protocol	Long Protocol	
	N = 29	N = 29	
**Oocyte Count**			0.001
Mean ± Std	5.60 ± 3.54	10.48 ± 6.66	
Median	5.00	8.00	
Range (min-max)	15.00 (1.00–16.00)	30.00 (3.00–33.00)	
**Fertility Rate**			0.709
Mean ± Std	3.45 ± 1.79	3.62 ± 2.01	
Median	3.00	3,00	
Range (min-max)	7.00 (1.00–8.00)	10.00 (1.00–11.00)	
**Pregnancy Rate**			0.009
Mean ± Std	157.17 ± 250.58	371.47 ± 365.66	
Median	61.75	216.20	
Range (min-max)	1009.99 (0.01–1010.00)	1176.96 (0.10–1177.66)	

Unlike the fertilization rate, the pregnancy rate variable found a statistically significant difference between the short and long group variables. In the short group, the average pregnancy rate was 157.17 ± 250.58, while in the long group, the average was 371.47 ± 365.66.

## 4 Discussion

### 4.1 Oocytes count in the short and long groups

This study found a significant difference in the oocytes count which is in line with the Ramalingam study in 2016 that GnRH antagonists were preferred over GnRH agonists for short protocols [11]. GnRH antagonists inhibit the rapid release of gonadotropins (only a few hours) by competitively occupying the pituitary GnRH receptor. While in the long protocol there was a decreasing of LH and FSH secretion due to down-regulation of GnRH receptors and pituitary desensitization. Decreased of LH and FSH suppresses the growth and ovulation of ovarian follicles, resulting in low circulating levels of estradiol and progesterone [11].

The number of oocytes in long protocol group has an average of 9.30 ± 6308. The agent used in the long protocol was a GnRH agonist. This is in line with the research by Annalisa Racca in 2020, which stated that the use of GnRH agonists strongly suppresses endogenous gonadotropin secretion during the early follicular phase, allowing antral follicles to grow in accordance with the exogenous gonadotropin response to reach mature follicles. GnRH agonists also increase the number of mature follicles and oocytes for embryo transfer [12].

From the above discussion, the main difference between the short and long protocols are the long protocol have two distinct stages of down-regulation and stimulation. Beside, in short protocol, the patient goes straight to the stimulation stage. The advantage of short protocol is that fewer drugs are used because it does not go through a down-regulation process. In addition, the risk of ovarian hyper stimulation syndrome (OHSS) is also lower [11]. The long protocol has several disadvantages for patients, the length of the stimulation process, the increasing of incidence possibility of ovarian hyperstimulation syndrome (OHSS), in addition to side effects such as bleeding, cyst appearance, and headaches that last longer than the short protocol. Therefore, the use of GnRH antagonists is considered to prevent the occurrence of LH surge in assisted reproductive technology [10,13].

In adenomyotic lesions found the presence of GnRH receptors, the use of GnRH agonists in the treatment process is considered to have antiproliferative, hypoestrogenic effects, reduce angiogenesis and inflammatory reactions that will induce apoptosis in cases of adenomyosis [14]. The presence of anti-proliferative, hypoestrogenic effects resulting from the administration of GnRH agonists is thought to be involved in the regression of adenomyosis in reducing the size of the uterus thereby suppressing the symptoms of adenomyosis [14].

### 4.2 Fertilization rate in the short and long groups

The average fertilization rate in the short group was 3.50 ± 1.881, while the long protocol was 3.57 ± 1.863. Although this difference was not statistically significant (p > 0.05). This is in line with Costello study in 2011 that adenomyosis did not affect the IVF program [8]. Factors that influence the incidence of infertility in adenomyosis are the increase of interleukin (IL-1β) and CRH expression. This suggests the involvement of endometrial inflammatory pathways that can increase free radical metabolism by releasing reactive oxygen by macrophages. It also triggers changes in the expression of pro-oxidant and antioxidant enzymes in the endometrium, which can affect hormone production and ovarian reserve. This can lead to amenorrhea and infertility [15].

Peritoneal fluid of patients with endometriosis found prostaglandins, levels of activated macrophages, tumor necrosis factor (TNF) ±, IL-1β, and increased proteases. It is known to affect the quality and function of the oocyte [13,16]. Meanwhile, in the case of adenomyosis, it is not known that it can affect both the quality and function of the oocytes.

### 4.3 Pregnancy rate in short and long group

This study showed a significant difference in pregnancy rates between the two study groups (p < 0.05). A higher pregnancy rate was found in the long protocol group (371.47 ± 365.66 compared to 157.17 ± 250.58). Consistent with these results, a retrospective study of 5662 IVF cycles reported a significantly higher clinical pregnancy rate in the long protocol group (p < 0.05). The study also explained that the difference in pregnancy rates was influenced by age [17].

In general, long protocols of GnRH agonists produced better effects than short protocols of GnRH antagonists, as indicated by clinical pregnancy rates (higher β-hCG levels). Some other reason the longer protocol is more often used in this case is to produce better follicular synchronization because it starts in the mid-luteal phase which allows an increase in both of size and harvested oocytes count, beside the effect of lower serum LH levels make qualified endometrium in the embryo implantation process. Limitation of this study is the pregnancy rate not assessed based on the take home baby but the level of β-hCG. In addition, there is no consensus on the cut off point of β-hCG which is considered to predict a pregnancy that is going well.

Although the long-term GnRH agonist protocol is considered superior to the short-term protocol, a prospective study by Hou et al. (2020) demonstrated a negative effect of adenomyosis on IVF success. A longer (ultra-long) GnRH agonist protocol is required to obtain more optimal IVF results. Hou et al. found that the clinical pregnancy rate was increased by 92.5% in adenomyosis patients who underwent an ultra-long protocol of GnRH agonists versus a long protocol (OR: 1.925; 95% CI: 1.137–3.250; p:0.015) [6]. Pregnancy in adenomyosis can occur in good condition of the uterus, and hyper peristaltic uterine contractions [6].

## 5 Conclusion

In conclusion, there were differences in the oocytes count and pregnancy rates in the short and long protocols, while there was no difference in fertilization rates between the two.

## Ethical approval

The study was approved by the Research Ethics Committee of Dr. Hasan Sadikin Hospital, Bandung, Indonesia, Nomor: LB.02.01/X.6.5/159/2022.

## Sources of funding

The authors declares no funding resources.

## Authors' contributions

1. Protocol/project development.

2Data collection or management.

3. Data analysis.

4Statistical analysis.

5. Literatur Search.

6. Manuscript writing/editing.

DT ^1,2,3,4,5,6^

AP ^1,2,3,4^

SDAR ^1,2,4^

ASN ^5,6^

## Registration of research studies

1. Name of the registry: http://www.researchregistry.com
2. Unique Identifying number or registration ID: researchregistry8088
3. Hyperlink to your specific registration (must be publicly accessible and will be checked):https://www.researchregistry.com/browse-the-registry#home/registrationdetails/62ce57b66d71dd0020aff8cd/

## Guarantor

The guarantors of this study is Dian Tjahyadi as the first author.

## Consent

N/A as this study does not involve patient participation.

### Availability of data and materials

The datasets used and/or analyzed during the current study are available from the corresponding author on reasonable request.

## Declaration of competing interest

The authors declare that they have no conflicts of interest.

## Abbreviations

IVF =: In-Vitro Fertilization
FET =: Frozen Thawed Embryo Transfer
AMH =: Anti Mullerian Hormone
ICSI =: intracytoplasmic sperm injection
AFC =: Antral Follicle Count

## References

[1] Campo S., Campo V., Benagiano G. (2012).

[2] Yu O., Schulze-Rath R., Grafton J., Hansen K., Scholes D., Reed S.D. (2020). Adenomyosis incidence, prevalence and treatment: United States population-based study 2006–2015. Am. J. Obstet. Gynecol..

[3] Wiweko B., Legiantuko A., Kemal A., Pratama G., Situmorang H., Sumapraja K. (2016). The outcome on conservative surgical treatment of adenomyosis. Indonesian Journal of Obstetrics and Gynecology.

[4] Xiao Y., Li T., Xia E., Yang X., Sun X., Zhou Y. (2013). Expression of integrin β3 and osteopontin in the eutopic endometrium of adenomyosis during the implantation window. Eur. J. Obstet. Gynecol. Reprod. Biol..

[5] Fischer C.P., Kayisili U., Taylor H.S. (2011). HOXA10 expression is decreased in endometrium of women with adenomyosis. Fertil. Steril..

[6] Hou X., Xing J., Shan H., Mei J., Sun Y., Yan G. (2020). The effect of adenomyosis on IVF after long or ultra-long GnRH agonist treatment. Reprod. Biomed. Online.

[7] Lin J., Sun C., Zheng H. (2000). Gonadotropin-releasing hormone agonists and laparoscopy in the treatment of adenomyosis with infertility. Chin. Med. J..

[8] Costello M.F., Lindsay K., McNally G. (2011). The effect of adenomyosis on in vitro fertilisation and intra-cytoplasmic sperm injection treatment outcome. Eur. J. Obstet. Gynecol. Reprod. Biol..

[9] Mathew G., Agha R., Albrecht J., Goel P., Mukherjee I., Pai P. (2021). Strocss 2021: strengthening the reporting of cohort, cross-sectional and case-control studies in surgery. International Journal of Surgery Open.

[10] Depalo R., Jayakrishan K., Garruti G., Totaro I., Panzarino M., Giorgino F. (2012). GnRH agonist versus GnRH antagonist in in vitro fertilization and embryo transfer (IVF/ET). Reprod. Biol. Endocrinol..

[11] Ramalingam M., Durgadevi P., Mahmood T. (2016). In vitro fertilization. Obstet. Gynaecol. Reprod. Med..

[12] Racca A., Drakopoulos P., Neves A.R., Polyzos N.P. (2020). Current therapeutic options for controlled ovarian stimulation in assisted reproductive technology. Drugs.

[13] Sanchez A.M., Vanni V.S., Bartiromo L., Papaleo E., Zilberberg E., Candiani M. (2017). Is the oocyte quality affected by endometriosis? A review of the literature. J. Ovarian Res..

[14] Khan K.N., Kitajima M., Hiraki K., Fujishita A., Sekine I., Ishimaru T. (2010). Changes in tissue inflammation, angiogenesis and apoptosis in endometriosis, adenomyosis and uterine myoma after GnRH agonist therapy. Hum. Reprod..

[15] Szubert M., Koziróg E., Olszak O., Krygier-Kurz K., Kazmierczak J., Wilczynski J. (2021). Adenomyosis and infertility—review of medical and surgical approaches. Int. J. Environ. Res. Publ. Health.

[16] Cheong Y., Shelton J., Laird S., Richmond M., Kudesia G., Li T. (2002). IL-1, IL-6 and TNF-α concentrations in the peritoneal fluid of women with pelvic adhesions. Hum. Reprod..

[17] Ou J., Xing W., Li Y., Xu Y., Zhou C. (2015). Short versus long gonadotropin-releasing hormone analogue suppression protocols in IVF/ICSI cycles in patients of various age ranges. PLoS One.

